# Supplementary material for: A Likelihood Approach for Real-Time Calibration of Stochastic Compartmental Epidemic Models
Source: PLoS Comput Biol. 2017 Jan 17;13(1):e1005257. doi: 10.1371/journal.pcbi.1005257 (PMC5240920; doi:10.1371/journal.pcbi.1005257)
Supplement: S1 File — (TAR.GZ) [file pcbi.1005257.s014.tar.gz › HSPH_Online-SI-Revision/EnKF07/n100-severe/n100-severe_EnKF_table0.pdf]

1	R0	{1.65278, 9.06793}	{2.63391, 13.0752}	{{2.14789, 3.69261}, {3., 19.1865}}	{59.3625, 78.2948}	inf	9005.	1949.93	{323.694, 2202.98}	83.2645	0.901816	newly	6768.	1431.64	{179., 2195.}	85.0591	1.00968	Reff	0.958398	0.882812	{0.367685, 2.14786}	52.3206	ar	66 342.	181 169.	{161 773., 244 425.}	173.084
2	R0	{1.4321, 3.80644}	{2.37093, 11.4318}	{{0.268956, 4.45355}, {3., 16.5229}}	{88.4882, 200.329}	inf	4783.	3182.64	{584.241, 6548.01}	75.8255	0.513616	newly	7700.	3040.48	{310., 3578.}	96.5291	0.911693	Reff	0.875729	0.553479	{0.250351, 0.667854}	53.318	ar	54 217.	177 490.	{165 804., 184 333.}	227.37
3	R0	{1.68388, 4.76028}	{2.30967, 7.75109}	{{0.0185129, 2.17576}, {3., 11.4001}}	{71.7102, 87.7779}	inf	8327.	6440.94	{2410.4, 7965.42}	76.4044	0.392042	newly	10 200.	6074.7	{1465., 8964.}	81.6518	0.544829	Reff	0.78489	0.608796	{0.0185128, 0.788826}	39.0174	ar	68 299.	188 476.	{173 250., 204 244.}	175.958
4	R0	{1.66014, 6.95383}	{2.57852, 5.56847}	{{1.54807, 3.60546}, {3., 11.5804}}	{58.4945, 49.1963}	inf	9062.	6911.09	{269.725, 7262.21}	101.617	0.709479	newly	8732.	6624.36	{337., 11 749.}	89.8731	0.692229	Reff	0.943906	0.625987	{0.293358, 0.699003}	49.7646	ar	66 820.	182 240.	{162 492., 224 788.}	172.733
5	R0	{1.69984, 11.2038}	{2.66382, 12.9756}	{{2.19163, 3.63139}, {3., 19.3222}}	{56.71, 59.6582}	inf	9747.	1826.87	{488.072, 5256.57}	81.6277	0.887855	newly	5980.	1472.13	{323., 4024.}	77.4085	0.84561	Reff	0.958166	0.959106	{0.453708, 2.11781}	54.7001	ar	68 462.	181 514.	{159 206., 237 334.}	165.131
6	R0	{1.65876, 6.66217}	{2.56342, 5.50944}	{{1.55465, 3.57966}, {3., 11.8202}}	{57.6044, 49.8203}	inf	9053.	8707.99	{711.569, 7331.03}	113.388	0.594676	newly	9118.	8917.31	{693., 30 184.}	104.231	0.597894	Reff	1.0042	0.654487	{0.338923, 0.79904}	53.9837	ar	67 105.	187 510.	{164 160., 267 936.}	179.427
7	R0	{1.69966, 5.89996}	{2.34104, 11.3824}	{{0.328021, 4.29333}, {3., 16.4975}}	{62.0321, 104.833}	inf	10 219.	3535.19	{1088.27, 7190.82}	75.0762	0.678475	newly	11 408.	3428.99	{507., 4222.}	93.8244	0.969489	Reff	0.93415	0.585968	{0.294709, 0.706256}	49.5458	ar	69 396.	178 373.	{167 102., 187 842.}	157.036
8	R0	{1.53428, 6.3734}	{2.63601, 6.28623}	{{1.7503, 3.25031}, {3., 8.58247}}	{71.8076, 37.9006}	inf	6765.	2907.97	{665.26, 5681.53}	81.9001	0.652619	newly	7091.	3865.56	{385., 6596.}	96.4741	0.770822	Reff	0.973639	0.44642	{0.125839, 0.838944}	60.1922	ar	59 855.	179 044.	{167 259., 189 615.}	199.13
9	R0	{1.58939, 3.48608}	{2.07752, 7.68168}	{{0.0285328, 2.35007}, {3., 12.0269}}	{81.0901, 137.814}	inf	7643.	6253.72	{2937.19, 7588.44}	66.0472	0.312648	newly	13 814.	5554.12	{1690., 9961.}	73.0969	0.604535	Reff	0.959229	0.506799	{0.00813869, 0.778287}	50.4066	ar	62 575.	183 149.	{171 168., 202 177.}	192.688
10	R0	{1.58528, 5.5593}	{1.93649, 9.94757}	{{0.0536778, 4.11887}, {3., 14.5689}}	{85.4896, 89.8394}	inf	7290.	1090.75	{141.314, 1731.68}	88.7987	1.11111	newly	8668.	784.35	{94., 931.}	92.962	1.38062	Reff	0.933207	0.381124	{0.00955275, 0.902581}	66.611	ar	63 039.	166 055.	{161 076., 171 271.}	163.416
11	R0	{1.58305, 4.62721}	{2.35187, 11.6108}	{{0.255677, 4.34803}, {3., 16.4843}}	{72.2448, 153.255}	inf	7294.	3382.14	{990.679, 6891.91}	69.955	0.549344	newly	9915.	3203.68	{464., 3812.}	94.2213	0.924256	Reff	0.910428	0.566753	{0.24164, 0.694823}	50.6073	ar	63 222.	177 299.	{166 572., 183 096.}	180.438
12	R0	{1.70234, 9.34626}	{2.65831, 12.9323}	{{2.1457, 3.58541}, {3., 18.9535}}	{56.1562, 73.8131}	inf	9412.	1334.65	{318.779, 1976.65}	86.2181	1.0521	newly	6925.	907.74	{151., 1501.}	87.3938	1.14493	Reff	0.976973	0.884093	{0.464134, 2.14568}	44.3628	ar	68 213.	176 503.	{161 553., 243 132.}	158.753
13	R0	{1.68556, 9.74954}	{2.63318, 12.8512}	{{2.13783, 3.64492}, {3., 19.0976}}	{56.2201, 69.1016}	inf	9667.	2192.04	{262.5, 2354.79}	80.822	0.918027	newly	6771.	1833.27	{153., 7302.}	79.4194	0.967784	Reff	0.938014	0.907255	{0.336332, 2.12534}	59.8896	ar	68 426.	184 377.	{160 582., 243 092.}	169.455
14	R0	{1.59876, 9.78897}	{2.67314, 13.2825}	{{2.15514, 3.82128}, {3., 19.4449}}	{67.2011, 70.8577}	inf	7640.	1341.51	{326.458, 1161.21}	88.911	1.00214	newly	5391.	890.44	{155., 1242.}	89.783	1.08838	Reff	1.0474	0.855017	{0.350026, 2.15512}	48.4809	ar	63 553.	177 119.	{161 177., 245 112.}	178.695
15	R0	{1.43577, 5.12819}	{2.21476, 7.29713}	{{0.342794, 2.3774}, {3., 10.9883}}	{80.8382, 69.0719}	inf	5339.	2167.69	{264.519, 1927.17}	95.3794	0.81689	newly	6966.	1667.69	{370., 2029.}	92.469	1.01319	Reff	0.97842	0.461521	{0.0790235, 0.759901}	56.7638	ar	53 259.	170 277.	{161 259., 190 600.}	219.715
16	R0	{1.58866, 8.73191}	{1.92, 10.2324}	{{0.17345, 4.12843}, {3., 14.8172}}	{82.9004, 49.3019}	inf	7934.	2100.33	{135.764, 1361.45}	98.7853	1.06019	newly	6295.	1345.88	{57., 1911.}	92.1782	1.14491	Reff	1.05624	0.499706	{0.0241232, 1.1576}	58.8247	ar	64 349.	171 744.	{163 520., 181 136.}	166.894
17	R0	{1.43012, 3.8163}	{2.39813, 11.7802}	{{0.612557, 4.51126}, {3., 17.1012}}	{84.9387, 208.682}	inf	5239.	2648.05	{210.476, 5334.92}	80.9557	0.745239	newly	8949.	2804.77	{90., 3562.}	98.6628	1.20927	Reff	0.975428	0.538923	{0.150548, 0.668754}	55.73	ar	53 353.	174 737.	{164 009., 190 698.}	227.511
18	R0	{1.47965, 5.16472}	{2.39582, 6.79608}	{{0.464023, 1.21236}, {3., 18.8522}}	{90.6997, 122.004}	inf	5515.	10 371.6	{1354.42, 18 033.4}	107.582	0.255607	newly	7086.	13 405.2	{1572., 23 091.}	142.667	0.425697	Reff	0.947154	0.563438	{0.0779845, 1.0028}	49.9567	ar	56 086.	187 567.	{164 355., 224 271.}	234.428
19	R0	{1.57191, 5.2314}	{2.35089, 6.43707}	{{0.412629, 1.38405}, {3., 18.538}}	{81.9134, 114.184}	inf	6751.	9760.96	{986.586, 14 033.4}	100.033	0.341631	newly	7954.	11 104.1	{1362., 11 791.}	124.274	0.468604	Reff	0.829937	0.447972	{0.0889287, 0.679594}	54.4135	ar	63 119.	185 547.	{163 509., 226 714.}	193.963
20	R0	{1.54435, 5.32911}	{2.63986, 6.33114}	{{1.75191, 3.20389}, {3., 8.67576}}	{70.9366, 47.5029}	inf	6968.	2687.01	{816.866, 3643.8}	77.3426	0.614556	newly	8356.	3633.4	{550., 4385.}	93.1774	0.787479	Reff	0.898704	0.437319	{0.176869, 0.704854}	58.1778	ar	61 870.	175 323.	{165 229., 185 616.}	183.373
21	R0	{1.57932, 4.90031}	{2.35635, 11.4824}	{{0.406454, 4.33927}, {3., 16.6529}}	{71.5718, 139.275}	inf	8141.	3391.98	{954.426, 6897.32}	73.4588	0.608506	newly	10 527.	3329.76	{428., 4297.}	94.7628	0.957117	Reff	0.930156	0.578094	{0.307381, 0.687809}	50.3224	ar	63 595.	177 451.	{166 147., 186 984.}	179.033
22	R0	{1.47044, 4.07694}	{2.25521, 7.09772}	{{0.483974, 1.13051}, {3., 19.3125}}	{89.5023, 149.995}	inf	5758.	6930.25	{530.316, 6568.26}	76.7985	0.41892	newly	9422.	7763.22	{656., 13 085.}	90.3819	0.593485	Reff	0.991724	0.504171	{0.0629214, 0.807738}	55.8944	ar	55 895.	181 064.	{162 295., 223 910.}	223.936
23	R0	{1.64997, 3.34546}	{2.05866, 5.89808}	{{0.755, 3.24777}, {3., 8.97304}}	{66.7862, 80.5966}	inf	6829.	1719.53	{313.29, 1660.35}	88.4179	0.873277	newly	10 882.	1622.24	{196., 1535.}	91.0724	1.13409	Reff	0.75727	0.335778	{0.0888853, 0.496}	62.7248	ar	66 404.	168 699.	{160 928., 181 747.}	154.049
24	R0	{1.67405, 4.50254}	{2.23695, 4.94809}	{{1.62397, 3.94791}, {3., 6.75076}}	{46.6492, 29.6958}	inf	9359.	3536.39	{436.597, 6760.32}	85.6017	0.790093	newly	12 907.	3838.95	{346., 7204.}	89.2608	0.953325	Reff	0.908708	0.356428	{0.0673671, 0.633893}	65.7838	ar	68 017.	185 636.	{169 937., 245 673.}	173.5
25	R0	{1.50663, 3.1286}	{2.24006, 4.62682}	{{1.6278, 0.0897452}, {3., 6.79971}}	{59.1458, 69.5632}	inf	5849.	3750.07	{508.672, 8012.34}	90.7207	0.614585	newly	11 133.	4187.5	{435., 8499.}	87.6233	0.854754	Reff	0.89994	0.35819	{0.062462, 0.647339}	65.284	ar	58 823.	181 010.	{166 248., 229 952.}	208.316
26	R0	{1.69822, 6.34992}	{2.71301, 4.5016}	{{2.25491, 0.215425}, {3., 6.43276}}	{59.7559, 30.5428}	inf	9988.	5960.27	{3265.52, 9285.48}	70.1554	0.467768	newly	10 139.	9269.24	{2421., 11 890.}	87.3139	0.44851	Reff	0.906629	0.652642	{0.28131, 0.762646}	44.5284	ar	69 816.	182 818.	{167 840., 199 015.}	161.856
27	R0	{1.55869, 4.92988}	{2.31613, 6.69331}	{{0.430313, 1.28927}, {3., 19.068}}	{83.2665, 124.79}	inf	7340.	8430.51	{306.655, 19 357.9}	88.0887	0.444274	newly	9500.	10 054.4	{393., 13 193.}	101.489	0.576398	Reff	0.908124	0.475188	{0.0636221, 0.992051}	56.6207	ar	62 344.	182 303.	{160 211., 226 584.}	192.415
28	R0	{1.46085, 6.06899}	{2.75393, 4.39988}	{{2.25695, 0.158085}, {3., 6.23013}}	{88.5156, 30.3168}	inf	5497.	5345.3	{2261.09, 9733.72}	73.1476	0.311673	newly	5894.	8831.03	{2221., 16 331.}	122.389	0.389985	Reff	0.925463	0.68885	{0.319522, 1.05131}	42.2261	ar	55 484.	180 699.	{164 734., 196 754.}	225.677
29	R0	{1.63017, 6.25164}	{2.38384, 6.76268}	{{0.42847, 1.3343}, {3., 18.7229}}	{77.1455, 107.419}	inf	8283.	9293.24	{525.1, 19 779.5}	91.3534	0.44293	newly	8506.	11 029.	{681., 18 046.}	120.802	0.533631	Reff	0.896577	0.463252	{0.0669632, 0.939176}	57.9247	ar	66 231.	183 370.	{160 005., 225 826.}	176.865
30	R0	{1.45173, 5.68165}	{1.97927, 10.0472}	{{0.147137, 4.141}, {3., 14.7426}}	{93.5754, 88.2207}	inf	5301.	1646.89	{399.973, 1486.92}	86.2734	0.802252	newly	6273.	1384.03	{171., 2046.}	90.4516	1.06651	Reff	0.953496	0.397412	{0.0302456, 0.77429}	67.3388	ar	55 280.	166 491.	{159 113., 170 752.}	201.178
31	R0	{1.55808, 2.77316}	{2.36923, 4.97783}	{{1.61643, 3.86776}, {3., 6.77077}}	{52.0609, 79.5004}	inf	4924.	3682.3	{679.413, 5738.63}	90.4525	0.571905	newly	9080.	4183.26	{475., 7444.}	92.0403	0.825146	Reff	0.782265	0.378411	{0.0718324, 0.62777}	61.8915	ar	60 940.	190 194.	{172 612., 246 932.}	212.1
32	R0	{1.56797, 3.90327}	{2.01342, 7.81614}	{{0.034248, 2.34105}, {3., 11.9698}}	{83.1788, 120.143}	inf	6996.	6040.77	{2194.87, 6893.68}	82.6971	0.377947	newly	11 191.	4965.16	{1284., 9035.}	78.2632	0.628943	Reff	0.933679	0.48652	{0.010982, 0.776007}	51.1569	ar	62 228.	181 836.	{170 006., 191 496.}	192.209
33	R0	{1.70027, 5.43261}	{2.13035, 7.88134}	{{0.36122, 2.12658}, {3., 11.7097}}	{72.9398, 73.0479}	inf	8804.	5933.93	{2082.96, 6728.}	80.6237	0.460809	newly	10 094.	4786.11	{1336., 7361.}	79.6898	0.606513	Reff	0.817558	0.467554	{0.0736449, 0.696011}	43.4301	ar	68 798.	183 729.	{170 861., 193 941.}	167.056
34	R0	{1.61668, 6.86192}	{2.66595, 13.2638}	{{2.13652, 3.6341}, {3., 18.8786}}	{64.9025, 117.396}	inf	8157.	2219.53	{608.324, 2743.62}	87.433	0.857675	newly	7767.	1399.85	{275., 2276.}	87.8474	1.04412	Reff	0.928411	0.973294	{0.528737, 2.1365}	41.898	ar	64 693.	183 163.	{164 751., 246 703.}	183.126
35	R0	{1.55955, 3.45071}	{2.15393, 6.0258}	{{0.816845, 3.27941}, {3., 9.30813}}	{72.2349, 79.7538}	inf	6759.	1194.57	{329.533, 1448.09}	86.3707	0.940858	newly	11 360.	1075.21	{346., 1321.}	91.0478	1.23129	Reff	0.876124	0.345239	{0.0915828, 0.495853}	65.7761	ar	61 783.	170 757.	{165 004., 185 768.}	177.683
36	R0	{1.65068, 4.91307}	{2.42534, 11.1693}	{{0.519297, 4.283}, {3., 16.7967}}	{65.0705, 133.333}	inf	9077.	3387.88	{979.965, 6614.15}	76.5612	0.68636	newly	12 338.	3447.	{429., 4632.}	94.5853	1.03888	Reff	0.997374	0.595519	{0.291387, 0.717666}	50.4089	ar	66 453.	177 593.	{166 791., 188 243.}	167.246
37	R0	{1.46131, 3.06714}	{2.14278, 6.16077}	{{0.864292, 3.33603}, {3., 9.42705}}	{76.6511, 103.252}	inf	5039.	1669.46	{440.431, 1428.41}	83.8067	0.74363	newly	9251.	1506.94	{374., 1549.}	89.3644	1.07796	Reff	0.841612	0.338312	{0.108602, 0.475454}	65.3948	ar	56 452.	166 682.	{160 481., 186 314.}	196.417
38	R0	{1.71034, 5.2546}	{2.34213, 11.2285}	{{0.0737971, 4.20548}, {3., 16.0301}}	{62.1707, 121.587}	inf	9467.	4146.38	{1431.77, 8625.24}	67.2937	0.528311	newly	11 210.	3681.8	{762., 4126.}	90.5909	0.837075	Reff	0.857787	0.579141	{0.0514334, 0.729845}	44.3858	ar	69 816.	180 198.	{169 724., 185 158.}	158.104
39	R0	{1.43565, 5.57989}	{2.776, 4.56758}	{{2.25741, 0.140084}, {3., 6.25952}}	{93.3621, 31.5401}	inf	5165.	4970.7	{2431.99, 6828.31}	61.2489	0.247359	newly	6041.	7703.1	{2340., 15 550.}	96.4539	0.348466	Reff	0.90825	0.717847	{0.35615, 0.973172}	42.5521	ar	54 407.	182 219.	{165 525., 199 772.}	234.919
40	R0	{1.40327, 4.00764}	{2.40425, 11.8885}	{{0.508686, 4.53539}, {3., 16.9058}}	{89.3774, 196.645}	inf	4452.	2795.53	{236.997, 4921.51}	79.6338	0.664342	newly	7170.	2774.89	{91., 3341.}	99.4537	1.1052	Reff	0.942464	0.518509	{0.166853, 0.616822}	57.2829	ar	51 976.	173 782.	{162 972., 180 945.}	234.351
41	R0	{1.63612, 4.26741}	{2.12603, 6.07384}	{{0.840532, 3.35809}, {3., 9.3811}}	{65.5322, 57.6778}	inf	7141.	1933.41	{337.715, 1486.26}	85.7782	0.865285	newly	9905.	1805.11	{306., 2504.}	88.2802	1.07315	Reff	0.840131	0.337156	{0.108162, 0.478653}	65.3079	ar	65 029.	170 926.	{163 907., 191 735.}	164.564
42	R0	{1.57426, 6.50271}	{2.20988, 7.29912}	{{0.314666, 2.34371}, {3., 10.9772}}	{71.9259, 50.7683}	inf	7623.	2611.4	{103.374, 1679.65}	103.803	1.06881	newly	7814.	1858.51	{126., 1688.}	98.5218	1.16367	Reff	0.952711	0.470044	{0.0742539, 0.804441}	54.7883	ar	62 522.	170 893.	{160 458., 181 903.}	173.333
43	R0	{1.37818, 5.37636}	{1.96248, 9.63104}	{{0.1533, 4.16128}, {3., 14.7887}}	{100.672, 88.6305}	inf	4296.	1723.37	{445.833, 1384.9}	82.1259	0.646995	newly	5382.	1373.69	{259., 1663.}	87.9954	0.915499	Reff	0.94794	0.476577	{0.0503173, 0.793558}	58.7647	ar	50 098.	168 416.	{161 522., 173 193.}	236.174
44	R0	{1.66606, 4.92641}	{2.0233, 7.65447}	{{0.033828, 2.3103}, {3., 12.0259}}	{76.7005, 83.9464}	inf	9374.	6620.08	{2498.43, 9972.64}	81.8061	0.447406	newly	12 427.	5223.78	{1425., 9650.}	74.2776	0.614894	Reff	0.958134	0.470285	{0.0107533, 0.780785}	51.1373	ar	67 066.	182 560.	{170 677., 202 774.}	172.209
45	R0	{1.623, 5.9449}	{2.58575, 5.49111}	{{1.54907, 3.57771}, {3., 11.7803}}	{61.3591, 48.8596}	inf	8468.	7929.7	{718.2, 6687.22}	108.952	0.591731	newly	9497.	8564.29	{377., 19 953.}	100.848	0.636065	Reff	0.985843	0.648428	{0.201284, 0.793058}	53.6554	ar	64 554.	185 688.	{164 116., 232 400.}	187.648
46	R0	{1.65235, 5.51234}	{2.2755, 7.05486}	{{0.459746, 1.24436}, {3., 19.2917}}	{75.1352, 121.956}	inf	8716.	7353.13	{722.072, 6531.76}	75.2813	0.450934	newly	10 647.	9560.13	{371., 21 448.}	84.6564	0.576333	Reff	0.988634	0.562089	{0.0634728, 0.996607}	49.1286	ar	66 509.	180 777.	{161 521., 225 267.}	171.809
47	R0	{1.62138, 8.82208}	{2.80421, 9.81346}	{{2.39292, 4.38983}, {3., 14.878}}	{72.952, 44.6365}	inf	8399.	1789.03	{143.765, 1546.79}	85.6871	0.951712	newly	6515.	1887.58	{59., 2460.}	86.8629	1.00144	Reff	0.936704	0.746726	{0.184841, 1.9673}	56.3455	ar	65 598.	176 288.	{161 437., 229 126.}	168.74
48	R0	{1.64073, 6.69261}	{2.58084, 5.6606}	{{1.55122, 3.59228}, {3., 11.7024}}	{59.8552, 49.8538}	inf	8727.	7662.52	{402.869, 7080.16}	108.492	0.633639	newly	8793.	7265.91	{216., 22 038.}	91.8113	0.637998	Reff	0.968375	0.649013	{0.345653, 0.716243}	51.7684	ar	66 438.	184 225.	{162 544., 231 070.}	177.289
49	R0	{1.65556, 3.796}	{2.23944, 5.02046}	{{1.61439, 3.93025}, {3., 6.77332}}	{48.6034, 33.7693}	inf	8332.	3744.24	{111.905, 6885.9}	88.6097	0.852076	newly	12 770.	3935.36	{103., 7698.}	90.3353	1.05856	Reff	0.845809	0.345237	{0.0377182, 0.641073}	64.3106	ar	66 575.	181 036.	{165 325., 229 774.}	172.306
50	R0	{1.56828, 3.65713}	{2.11636, 6.06958}	{{0.854944, 3.34056}, {3., 9.35918}}	{69.1736, 73.374}	inf	6216.	1820.56	{348.726, 1445.28}	83.6845	0.783807	newly	9733.	1690.26	{326., 2560.}	87.8231	1.04037	Reff	0.821841	0.350362	{0.110003, 0.534503}	63.3171	ar	62 761.	172 044.	{163 585., 191 352.}	174.126
